# A randomized controlled trial study of the acceptability, feasibility, and preliminary impact of SITA (SMS as an Incentive To Adhere): a mobile technology-based intervention informed by behavioral economics to improve ART adherence among youth in Uganda

**DOI:** 10.1186/s12879-020-4896-0

**Published:** 2020-02-24

**Authors:** Sarah MacCarthy, Zachary Wagner, Alexandra Mendoza-Graf, Carlos Ignacio Gutierrez, Clare Samba, Josephine Birungi, Stephen Okoboi, Sebastian Linnemayr

**Affiliations:** 1 Behavioral and Policy Sciences, RAND Corporation, 1776 Main Street, Santa Monica, CA USA; 2 Economics, Sociology, Statistics, RAND Corporation, Santa Monica, CA USA; 3 Pardee RAND Graduate School, Santa Monica, CA USA; 4 TASO Uganda, Kampala, Uganda; 5 Infectious Diseases Institute, Makerere University, Kampala, Uganda

**Keywords:** Behavioral economics, ART adherence, Youth, Uganda

## Abstract

**Background:**

Studies report serious adherence problems among youth (individuals age 15–24 years of age) in Uganda. Recent growth in mobile phone ownership has highlighted the potential of using text-based interventions to improve antiretroviral treatment (ART) adherence among Ugandan youth. We piloted a randomized controlled trial of a text-based intervention providing weekly real-time antiretroviral adherence feedback, based on information from a smart pill box, to HIV-positive Ugandan youth. In this paper, we report the acceptability, feasibility, and preliminary impact of the intervention.

**Methods:**

We randomized participants to a control group, or to receive messages with information on either their own adherence levels (Treatment 1 - T1), or their own adherence and peer adherence levels (Treatment 2 – T2). We conducted six focus groups from December 2016 to March 2017 with providers and youth ages 15–24, double coded 130 excerpts, and achieved a pooled Cohen’s Kappa of 0.79 and 0.80 based on 34 randomly selected excerpts.

**Results:**

The quantitative and qualitative data show that the intervention was deemed acceptable and feasible. After controlling for baseline adherence, the T1 group had 3.8 percentage point lower adherence than the control group (95% CI -9.9, 2.3) and the T2 group had 2.4 percentage points higher adherence than the control group (95% CI -3.0, 7.9). However, there was an increasing treatment effect over time for the T2 group with the largest effect towards the end of the study; a 2.5 percentage point increase in the initial 9-weeks that grows steadily to 9.0 percentage points by the last 9-weeks of the study. We find negative treatment effects for T1 in 3 of the 4 9-week intervals. This pilot study was not designed to detect statistically significant differences.

**Conclusions:**

Improving youth’s adherence by supplementing information about their adherence with information about the adherence of peers is a promising new strategy that should be further evaluated in a fully-powered study. Providing one’s own adherence information alone appears to have less potential.

**Trial registration:**

NCT02514356 07/30/2015.

## Background

There are over 1.3 million people in Uganda living with HIV, and although those age 10–24 only make up 33% of the population, they represent 50% of the country’s HIV/AIDS cases [1, 2]. Further, studies in Uganda report serious adherence problems among youth 15–24 years of age [3–7], with barriers including HIV-related stigma, treatment disruptions, caretaker delay in disclosure of HIV status, lack of clinical support [8], and limited access to treatment in rural areas [9]. A systematic review focusing on youth in sub-Saharan Africa (SSA) identified additional barriers to adherence, including treatment side-effects and forgetfulness, while also highlighting facilitators, such as peer and caregiver support as well as knowledge of their own HIV status [10].

Recent growth in mobile phone ownership among youth in resource-poor settings [11] has highlighted the potential of using text-based interventions to improve antiretroviral treatment (ART) adherence [12, 13]. Futher, the simple and low cost nature of text-based interventions are particularly appealing to address HIV in low resource settings, especially in contrast to other often time and cost intensive approaches (e.g., cognitive behavioral therapy based interventions). However, evidence supporting the effectiveness of text-based interventions is mixed [6, 14] and novel strategies to use phone-based messages are needed. Behavioral economics (BE) offers novel insights into systematic decision-making errors (‘biases’) that might contribute to suboptimal adherence, potentially offering a way to enhance the effectiveness of text-based interventions.

This pilot study tested approaches rooted in BE that use text messages to improve ART adherence among youth (ages 15–24). We focused on two well-documented BE biases that may be particularly relevant for youth. We designed one intervention component to address ‘optimism bias’—the tendency of individuals to overestimate their capabilities [15]. People tend to overestimate the likelihood of positive experiences and underestimate the likelihood of negative ones and youth in particular tend to be particularly overoptimistic [16]. Preliminary analyses of data from our previous studies found that adults overestimate their own capability to adhere to their medication; self-reported adherence was 91% on average, while electronically measured adherence was only 80%. Yet despite this poor performance, the large majority of patients (81%) believed that they would show 100% adherence in the subsequent month. This finding underscores the importance of feedback to make respondents aware of their true adherence level. Therefore, in our intervention we used text messages to give participants weekly feedback about their recent ART adherence to counter optimism bias.

We designed a second intervention component to leverage ‘reference dependence bias’—the tendency of individuals to want to equal or surpass the performance of their peers [17]. Peer comparison has been effective at improving health worker performance [18], voting behavior [19], and energy efficiency [20]. Youth are particularly attuned to the behavior of their peers, so leveraging this bias may be particularly effective with this age group. We used text messages to give individuals information about the adherence of a reference group of their peers and to show how their own adherence compared to the group’s adherence, with the hypothesis that this would lead the participants to try to equal or even surpass the adherence level of their peer group.

We based the text messages on data collected by Wisepill, a smartpill box device that electronically records when pills are removed from the container and sends these data to a study computer. One treatment intervention arm of the study was given only their own individual ART adherence levels (T1), and the second intervention arm was given both individual and group ART adherence levels (T2). The control arm received the usual standard of care as provided by the clinic, including any adherence support mechanisms. In this paper, we describe the results from our pilot study regarding the acceptability, feasibility, and preliminary impact of the intervention.

## Methods

Our quantitative [21] and qualitative methods [22] are described in detail elsewhere; here we provide a brief summary of the data collected and methods used.

### Quantitative study

Study population and recruitment: Study participants age 15–24 were recruited from The AIDS Support Organization (TASO) at Mulago clinic in Kampala, the capital of Uganda, and in the suburb of Entebbe during scheduled clinic visits. Eligibility criteria included: 1) knows own HIV status, has disclosed to caretaker (if minor); 2) in care at TASO for at least 3 months, intends to seek care from this facility for the next year, and not currently participating in another health-related study; 3) taking ART or co-trimoxazole; 4) has regular access to a cell-phone (at least 1 hour per day, 5 days a week); and 5) is not in boarding school (as mobile phones are often forbidden). Recruitment took place between August 2015 and February 2016.

Once recruited, participants were given a Wisepill device and told to begin using it immediately. Participant’s adherence was monitored via Wisepill for 2 months before announcing their assignment to the intervention or control arms of the study (technical specifications of the device can be found at https://www.wisepill.com/rt2000). We excluded recruited clients who demonstrated less than 20% adherence during this two-month period because it suggested they were not using the Wisepill device, and consistent use of the device was necessary to accurately measure adherence. Twenty-four of 179 initially recruited clients (13%) were excluded for falling below this 20% threshold. Once the 179 study participants were recruited, the study team randomly assigned them to one of the three study arms using a random number generator in Stata; this method assured that treatment assignment could not subsequently be tapered and hence avoids selection bias. Importantly, clients were not informed of their random assignment until after completing the baseline survey, thereby avoiding any selection bias that may have come about if allocation to a treatment arm would have influenced participants’s use of the Wisepill device.

Interventions: After this two-month monitoring period, we informed the remaining 155 participants of their randomly assigned group. In the control group (*n* = 59), participants received care as usual, including any adherence support offered in the clinic. Each of the interventions lasted for 9 months. In the first treatment intervention arm (T1; *n* = 40), clients received a weekly text informing them of their adherence level in the previous week as measured by the Wisepill device. This intervention was designed to provide feedback to counter the observed bias of overestimating one’s own adherence. In the second treatment intervention arm (T2; *n* = 56), clients received information about their own adherence as well as information about the adherence level of their peers in the intervention (see Appendix Table A1 for exact wording of the weekly messages). We sent out group adherence levels between 80% and 93% to make sure not to send out adherence information that may lead recipients to take their pills at clinically suboptimal levels, or group adherence that is so high as to be perceived as demotivating. While there is not one cut-off that clearly defines clinically meaningful adherence, we set this level at 80% mean adherence after consultation with the participating clinics’ medical staff. We also refrained from sending out adherence levels that may demotivate recipients, which was a concern we heard in the formative phase of the study. Using these two principles, we therefore each week first checked whether the person in the 65th percentile (based on the pre-intervention data, that was the percentile where usually participants fell in the desired adherence range) had an adherence level of at least 80% and under 93%; if this was not the case, we used a random number generator set at between 80 and 93% and sent out that number as that week’s group adherence level.

Quantitative data: We used two sources of quantitative data: 1) two waves of participant surveys (baseline and 9-month follow-up) to collect demographics and beliefs/behaviors related to HIV treatment; 2) data recorded by the Wisepill device, which recorded the number of doses taken by each participant during the study. Baseline surveys were conducted between October 2015 and April 2016; endline surveys began in July 2016 and ended in February 2017.

Analysis of preliminary impact: To assess the preliminary impact of the two modes of text-based interventions, we used an intention to treat framework. Specifically, we coded respondents according to their original assignment rather than whether they actually received or viewed the messages. We used linear regression to compare adherence in the intervention and control groups. We estimated average treatment effects with all post-intervention periods pooled using the following regression model:

$$ Adherenc{e}_{it}={\beta}_0+{\lambda}_1T{1}_i+{\lambda}_2T{2}_i+\alpha BaseAdherenc{e}_i+{\epsilon}_{it,} $$

where *Adherence* is the average adherence over the entire 9 months for individual *i* in week *t*, *T*1 and *T*2 are indicators for T1 or T2 assignment, *BaseAdherence* is an indicator for the adherence level in the baseline period, and *ϵ* is an idiosyncratic error term. The *λ* s represent the average treatment effects of the two interventions over the 9 study months relative to the control group.

In addition, we assessed how treatment effects evolved over time by splitting the 36 -week study into four 9-week intervals and estimated the following equation.

$$ Adherenc{e}_{it}={\beta}_0+\sum \limits_{w=1}^4{\lambda}_wT{1}_i\times In{t}_{wt}+\sum \limits_{w=1}^4{\gamma}_wT{2}_i\times In{t}_w+{\epsilon}_{it} $$

Where the *T*1_*i*_ × *Int*_*w*_ and *T*2_*i*_ × *Int*_*w*_ are interaction terms between treatment assignment and time interval (relative to the difference in the baseline period (*w* =0). The coefficients, *λ*_*w*_ and *γ*_*w*_, represent the treatment effect in each time interval. We clustered standard errors by individual to account for potentially correlated data in the error term.

Although we estimate standard errors and confidence intervals, this is a pilot study; it was not powered to detect statistically significant treatment effects. We estimated that our sample size would allow us to detect a 6 percentage point effect in mean adherence between the interventions and the control group with 80% power (2-tailed t-test test).

### Qualitative study

Qualitative data: We conducted six exit focus groups (FG) - 1 with providers and 5 with youth between December 2016 and March 2017 to evaluate satisfaction with the intervention arms and to identify areas for improvement (Table 1).

**Table 1:** Characteristics of Exit Focus Groups

Type of FG participant	Eligibility criteria	Participants
Providers (*n* = 1)	Requested participation from all providers who had frequent contact with youth enrolled in SITA.	7 participants: 3 counselors, 1 drug dispenser/pharmacist, 1 client representative, 2 study coordinators
Patient - Youth (*n* = 4)	Participants in either of the treatment arms	Entebbe T1: 5 youth age 18+ including 2 female, 3 male age 18+ and 3 minors age < 18 including 2 female, 1 male
		Entebbe T2: 9 youth age 18+ including 4 male, 5 female
		Mulago T1: 9 youth age 18+ including 2 male, 7 female
		Mulago T2: 7 youth age 18+ including 1 male, 6 female
Patient - Minors (*n* = 1)	Participants in either of the treatment arms, but all below age 18	Mulago - minors only group: 5 minors below age 18 including 2 male, 3 female

For the provider FG, all providers with frequent patient contact were asked to participate in the FG. Patient FGs were divided between participants over 18 years of age and under 18 years of age. For all FGs, verbal consent was obtained (as requested by the study IRBs). Trained recruiters emphasized repeatedly that participation was voluntary, and that the same quality of services would be provided irrespective of whether the patient decided to participate. Providers were given the equivalent of $16 USD for their participation and patients received reimbursement of about $8 USD. All participants were also given lunch, a snack, and transportation money to the FG. These incentives were consistent with those provided for other studies at the same clinic.

All FGs were transcribed, translated from Luganda into English, and uploaded into the qualitative analysis software Dedoose. We used a directed content analysis approach: our relevant research provided guidance for identifying the intial themes (e.g., acceptability of receiving individual versus group adherence information) while also providing flexibility for additional themes to emerge (e.g., implementation challenges) [23]. Therefore we developed our preliminary codebook based on initial themes that we had anticipated, and revised it as two researchers jointly reviewed and coded a subset of transcripts, summing up to 130 excerpts. Revisions to the codebook were made; the final version included definitions for code with example text when helpful. The two researchers established inter-rater reliability on a set of 34 randomly selected exerpts based on a pooled Cohen’s Kappa of 0.79 and 0.80. The remaining interviews were single-coded, and any issues raised were discussed weekly. We complemented qualitative findings with summary notes from study staff.

Assessment of acceptability and feasibility: We drew on existing frameworks in the peer-reviewed literature to discuss core components of acceptability [24] and feasibility [25]. We describe acceptability based on the framework provided by Sekhon and colleagues [24] that assesses acceptability of an intervention based on cognitive and emotional responses to it. Tickle-Degnen [25] suggests determining feasibility based on four types of assessments: management, resource, scientific, and process. Adequate management of the study and adequate resources to conduct it are requirements for NIH funding. Here we focus on the scientific and process assessments (e.g., reliability of our measurement tools, adherence to study procedures) that determine the feasibility of large-scale implementation.

## Results

Demographic characteristics of participants are shown in Table 2. All participants had similar baseline adherence levels. Compared with the control group, participants in the two treatment arms were more likely to be male and have English as their first language. They also had a higher monthly income.

**Table 2:** Balance between Groups at Baseline

	Control	T1	T2
N	53	35	49
Baseline Adherence	0.81	0.79	0.79
Male	0.17	0.26	0.20
English Preferred Language	0.51	0.66	0.63
Can Read Newspaper	0.85	0.83	0.88
Completed Secondary Education	0.66	0.63	0.73
Currently Employed	0.51	0.43	0.45
Monthly Income (USD)	5.86	9.10	9.42
Has Bank Account	0.15	0.40	0.27

Notes: T1 = Received text messages with their own adherence information only, T2 = Received text messages with their own adherence as well as the adherence information of their peers

### Acceptability results

Intervention coherence – Do participants understand SITA? Qualitative data showed that participants understood the intervention protocols. They viewed SITA as an intervention to improve their adherence and emphasized the helpful role of Wisepill and receipt of adherence information.

Affective attitude – How do participants feel about SITA? Both quantitative and qualitative data show partiicpants had positive attitudes about SITA. In the follow-up survey data, 96.6% of participants reported that they would remain in the intervention if they had the choice (95.3% in the T1 group and 97.8% in T2), and 84.2% said there was nothing about SITA that they did not like (86.0% in T1 and 82.6% in T2). In the FGs, many participants in T2 reflected on enjoying the competition with their peers that was brought about by receiving information on peer adherence, while only one person from T1 mentioned liking the competition it caused from voluntarily sharing adherence information with peers. Further, several participants from both T1 and T2 felt that SITA boosted their morale and prompted them to take their ART medication.

Self-efficacy – Are participants able to perform the SITA activities? The intervention has two key components: use of the Wisepill device and use of the mobile phone to which SMS were delivered.

With respect to the Wisepill devise, the quantitiatve data shows that it recorded an 88% median level of adherence for participants, suggesting that most people used the Wisepill device to store their medication. Aditionally the survey showed that participants were generally very fond of the Wisepill device: more than half of participants reported that Wisepill was the part of the study they liked the most. The FGs found that many participants said their least favorite part of the study was having to give the device back. Further, participants in both treatment arms said that the Wisepill device was easy to move with and that the device itself helped as a reminder to take their medication. In the FGs, participants reported experiencing some challenges with the device–e.g., difficultly charging it; however, overall they appreciated its benefits. Anecdotal evidence from study staff noted some challenges in receiving Wisepill devices (e.g., fees required upon receipt at the airport) and preparing the devices for distribution (e.g., packaging the device along with the required cables, batteries, and plugs).

The second key protocol component was use of the mobile phone to which text messages were delivered. The quantitative data reveal exposure to the text messages was high, suggesting strong usage. Specifically, 75% of participants reported reading the messages every week, and 85% said they read them most weeks. Of note, wrong individual adherence information was sent on 82 occasions. Staff notes suggest that the errors were due to technological difficulties with SIM cards when trying to register them with the Ugandan phone service provider. In the FGs, participants reported that receiving inaccurate information undercut their desire to further improve their adherence. The study team subsequently resolved this problem in cooperation with the cell service provider.

Perceived effectiveness – Did participants think SITA was effective? Both the quantitative and qualitative data show that participants also thought SITA was effective. In the follow-up survey, 97.7% reported benefiting from being part of the SITA program (95.4% in the T1 group and 100% in the T2 group), and all participants reported that other youth at the clinic would benefit from being part of SITA. The FGs revealed that SITA consistently helped participants to take their medications on time. Further, participants noted that SITA’s impact extended beyond the specific act of taking their medication: in many ways, the program improved their overall outlook on life and generated renewed focus on their health.

Representative qualitative quotes that support the key findings on acceptability are shown in Table 3.

**Table 3:** Acceptability Results

Acceptability Component	Quotes
*Intervention Coherence*	“If […] you have the device, it helps you remember to take your drugs on time and also keeps drugs safe and helps to inspire you with the messages.” (Treatment Group 1)
*Affective Attitude*	Enjoying the Competition: “I would not love to lose SITA because it really changes a lot of people’s lives, and it has really changed my life because if I compare, sincerely speaking, my life now with the life backward or before SITA, it was really miserable in that I didn’t have anything to push me. But now with this project, there was competition, I was competing with the rest of my colleagues. I always wanted good marks from SITA, so it really encouraged me with all my friends. It really changed our lives. It was very wonderful.” (Treatment Group 1)
	Increased Morale: “When you have the Wisepill device, it comes to your mind that someone will know whether you took drugs or not so it was always boosting morale.” (Treatment Group 2)
	Helpful Reminders: “[SITA] was so good generally especially to us who used not to take our drugs well so it reminded us so much (Treatment Group 1)
*Self-Efficacy*	Use of Wisepill Device: “You can easily move with it without anyone knowing what you are carrying and it’s only you that knows. One can easily mistake it for a power bank and not mind about it. It was very good especially making it easy to move with it rather than moving with medication containers while making noise.” (Treatment Group 2)
	Use of SMS Messages: “I was eager to see the message because I very much wanted to see my percentage. Whenever, I saw my percentage for example like 30% or 50%, I would ask myself why it is 50%? I would ask myself, [and] it seemed here I skipped some minutes, so I am going to start to have to be punctual because poor adherence denied me a chance of the right percentage.” (Treatment Group 1)
*Perceived Effectiveness*	Taking medication on time: “According to me, [Wisepill] is good because I used to take my drugs on a daily basis but not on time. It used to motivate me and I would say, let me take drugs on time so that I score good marks because someone is monitoring me, so it always reminded me to take drugs on time. That was its merit.” (Treatment Group 1)
	Improved their overall quality of life: “It made me happy because before they gave it to me, I [thought] that even if I take the medication at the time I want, so long as I take, but for me it taught me that I have to take it on time... It made me set an alarm in my phone. If it rings I just know that I am missing something. In other words, it changed my life.” (Treatment Group 1)

### Feasibility results

Scientific assessments – Is SITA safe and standardized, and does it use valid measures? Study safety was established through the ethics approval processes at the RAND Corporation, TASO, and the Uganda National Council for Science and Technology. In addition, the study protocol was published in the clinical trials registry (ClinicalTrials.gov Identifier: NCT02514356 [21]). All measures of adherence are established using the electronic medication monitoring system Wisepill.

**Fig. 1:**
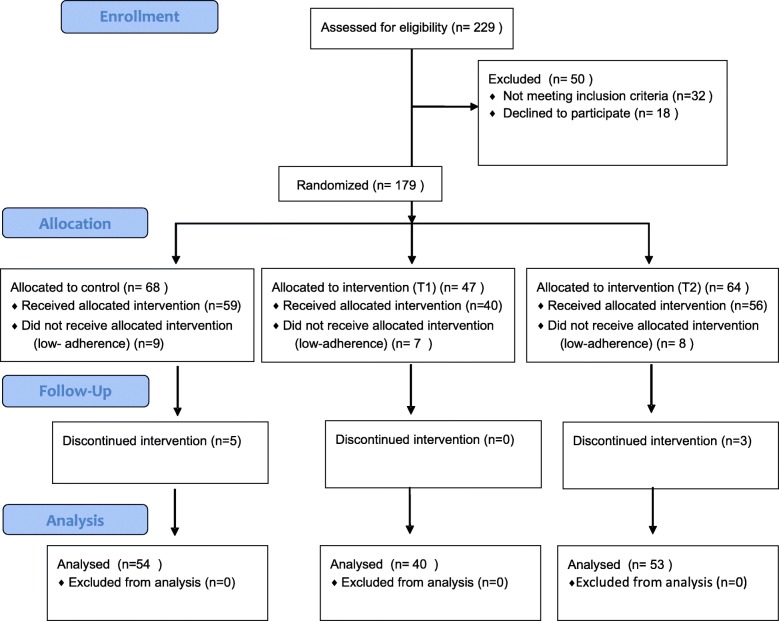
CONSORT Randomization Flow Diagram

Process assessment – What is the recruitment process and are study procedures followed? Figure 1 (CONSORT Flow Diagram) demonstrates that the eligibility criteria were feasible and not too narrow. Specifically, the quantitative data show that, of the 229 individuals approached, 32 were ineligible, most often because they did not have a working phone or did not meet the age requirement; 18 declined to participate in the study because they were not interested, or they provided no explanation. Staff notes also highlight that participants were concerned about potential disclosure of their HIV status (e.g., as a result of receiving calls from study staff associated with TASO, a known HIV service provider in the area). Despite these issues, target numbers were readily achieved. Of those recruited for the study, 24 failed to reach sufficient adherence in the pre-baseline period to warrant further study participation. Attrition was low, with only 8 (5 in control group, 3 in T2, and 0 in T1) of 155 participants lost to follow-up (5.16%).

### Preliminary impact results

**Fig. 2:**
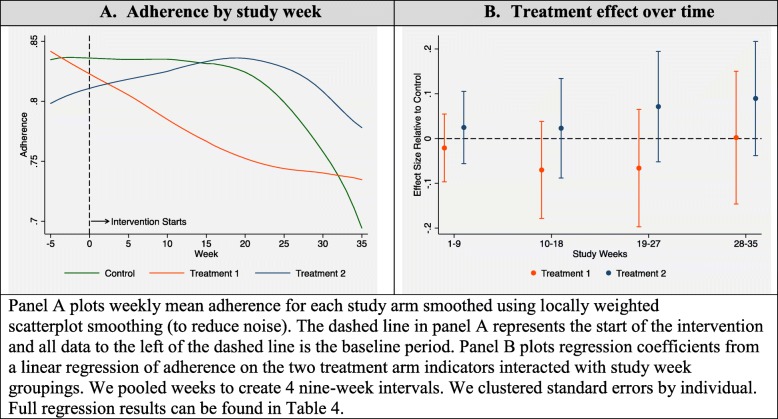
Intervention effects over time

The average effect over the entire 36 weeks was relatively small and statistically insignificant for both intervention groups. Adherence was 81.1% in the control group, 76.5% in T1 group, and 82.5% in the T2 group. After controlling for baseline adherence, the T1 group had 3.8 percentage point lower adherence than the control group (95% CI -9.9, 2.3) and the T2 group had 2.4 percentage points higher adherence than the control group (95% CI -3.0, 7.9). However, the average effect masks an increasing treatment effect over time for T2. To demonstrate this, Fig. 2a shows trends in adherence for the different study arms (smoothed using locally weighted scatterplot smoothing) over the 36 weeks and Fig. 2b shows treatment effects in each of the four 9-week intervals. Over the course of the study, adherence in the control group and in the T1 (own adherence information) group steadily dropped off. The control group began at over 80% adherence but fell to about 70% by the end of the study. The T1 group decreased from around 84 to 74%. In the T2 group, adherence increased initially and the subsequent drop off was less stark than in the other two groups. Adherence in the T2 group remained between 80 and 85% for the duration of the study. Figure 2b shows a 3 percentage point increase in adherence in the initial 9-weeks that grows to 9 percentage points by the last 9-weeks of the study. We found negative treatment effects for T1 in 3 of the 4 intervals.

Because this is a pilot study, our sample size is not large enough to give us sufficient power to detect statistically significant effects. However, the direction and magnitude of the treatment effects for T2 are promising; the T1 intervention shows no signs of impact. In other words, giving adolescents information about their own adherence (T1) does not appear to improve adherence, but giving them information about their own adherence relative to their peers (T2) shows promise for increasing adherence.

## Discussion

In this paper, we use established criteria to determine the acceptability, feasibility, and preliminary impact of a pilot intervention testing a novel approach to improve ART adherence based on behavioral economics and mobile health technologies. We provide evidence that SITA was acceptable and feasible among Ugandan HIV positive youth. Our analysis of preliminary impact suggests that giving individuals information on their own adherence does not improve adherence, but giving them information on their adherence relative to their peers could potentially improve their adherence. These results have important implications for the design of interventions aimed at increasing youth ART adherence.

Our analysis of acceptability focused on determining whether providers and youth considered SITA to be appropriate, beneficial, and not prohibitively burdensome. Both our quantitative and qualitative data show that youth understood SITA and felt positively about it. Thus, while many simple text message interventions have evolved to more advanced app development, several studies have shown that participants fail to take up or disengage from mobile health interventions over time [26–28]. Our pilot data suggest that our use of BE to address known biases (e.g., optimism bias and reference dependence bias) may enable the continued use of relatively simple technology and still improve ART adherence. This is especially important in resource poor settings such as Uganda, where ‘light touch’ interventions, meaning those requiring minimal financial or human resources, are needed that can still generate meaningful effect sizes.

The pilot study established the feasibility of sending text messages with information on a participant’s own adherence and adherence performance relative to peers. Receipt of group information has been shown to effectively improve other health behaviors, such as increased physical activity [29–31] and improved food choices [31–33], as well as minimizing alcohol use [34] and improving sexual health [35, 36]. Our pilot data suggests that it may also be used to improve ART adherence. Of note, some initial technical problems occurred with the use of Wisepill to relay the adherence information, however the issues were subsequently resolved. While use of Wisepill has been shown to be effective in other resource-poor settings [37, 38], our study highlights the need to maintain clear lines of communication with Wisepill distributors and local phone service providers, and to conduct routine data checks with participants to ensure accurate reporting of adherence. We also examined SITA’s safety, reliability of our measurement tools (e.g., the Wisepill device), feasibility of our recruitment process (size of eligible population, refusal and recruitment rates, and attrition), and adherence to study procedures (e.g., consistent use of the Wisepill device and retention of mobile phones).

Feedback from study staff highlighted several changes that could improve study implementation going forward. For example, they recommended identifying one individual to manage logistics associated with Wisepill, potentially helping to overcome some of the challenges in successfully receiving the devices in-country. Further, study staff noted that creating stronger contractual agreements with the phone company could facilitate communications when challenges arose (e.g., ensuring the phone company is willing to provide regular reports about the number of text messages sent, the number of messages bouncing back due to disconnected lines, etc.). Finally, study staff provided suggestions about increasing use of Wisepill among those participants who are hesitant to engage with technology. For example, participants who were concerned that the Wisepill device would signal their HIV status could be given potential responses when asked what the device was for (e.g., storage of vitamins, a power bank). Alternatively, if participants, especially those in more rural areas, referenced concerns about their ability to consistently charge their cell phones, it could be helpful to make additional batteries available, or send texts reminding them to charge their phone.

Findings from this pilot study suggest that giving individuals information on their own adherence does not improve adherence, but giving them information on their adherence relative to their peers may improve their adherence. A subsequent study at scale should be implemented to confirm these results and investigate whether the intervention works through the conceptual pathways hypothesized—countering optimism bias by providing own adherence information, and activating the power of social norms by providing information on the performance of the peer group.

### Limitations

The study has both limitations and strengths. First, we have limited data on participants who were excluded or dropped out during the intervention, minimizing our understanding of how such factors might affect future scale up of SITA. In particular, the sample was selected based on their use of Wisepill in the pre-baseline period (13% were excluded because they did not use the device consistently within the first 2 months of receiving the device) and some people declined to participate (9% of those eligible). Therefore, other strategies may be needed for individuals who are not comfortable using technology in this context. Second, we did not collect demographic information for the FG participants; thus despite our structured sampling frame, we cannot adequately compare similarities and differences between those who participated in the FGs compared to the intervention as a whole. Third, there was no clinical guidance to inform our definition of low adherers, so an arbitrary cutpoint was selected. Fourth, our study may also be limited by the Hawthorn effect associated with exposing all groups (including the control group) to the Wisepill device. The control could have increased their adherence in response to this device, because they knew their adherence was being monitored. Fifth, we had limited information on the adherence behavior of participants at baseline, and therefore we could not perform blocked randomization based on adherence characteristics. This would have likely improved the precision of our quantitative results. Finally, though this study was adequately powered for a pilot, a larger sample is needed to confirm our findings. A subsequent study at scale should be implemented to confirm these results and investigate whether the intervention works through the conceptual pathways hypothesized—countering optimism bias by providing own adherence information, and activating the power of social norms by providing information on the performance of the peer group. For example, T1 has a negative signed (though statistically insignificant) effect, which is counter-intuitive, and we do not have a good understanding of why this might occur. Future research will help understand if this is a real effect or an artifact of the small sample.

These limitations are balanced with significant strengths. Using both quantitative and qualitative data, our study assesses the acceptability, feasibility, and preliminary impact of a novel approach to using SMS messages to promote adherence. We used existing frameworks for our analysis of ‘acceptability’ and ‘feasibility,’ terms that are commonly used but rarely defined further. Finally, we further added insight from study staff to identify implementation challenges and suggest how future studies could overcome issues highlighted here.

## Conclusion

The intervention tested in this pilot study was found to be acceptable and feasible. The study provided preliminary evidence that giving youth information on their adherence relative to their peers can improve youth’s adherence. As the range of resource-intensive approaches to improving adherence grows, this simple and low cost approach warrants further investigation.

## Supplementary information

**Additional file 1: Table A1.** Messages sent to participants in each treatment group.

## Data Availability

The datasets generated and/or analysed during the current study are not publicly available due confidentiality concerns, but are available from the corresponding author on reasonable request.

## Abbreviations

TASO: The AIDS Support Organization
ART: Antoretroviral therapy
SSA: Sub-Saharan Africa
BE: Behavioral economics
FG: Focus group
HIV: Human immunodeficiency virus
SITA: SMS as an Incentive To Adhere
