# Supplementary material for: A randomized controlled trial study of the acceptability, feasibility, and preliminary impact of SITA (SMS as an Incentive To Adhere): a mobile technology-based intervention informed by behavioral economics to improve ART adherence among youth in Uganda
Source: BMC Infect Dis. 2020 Feb 24;20:173. doi: 10.1186/s12879-020-4896-0 (PMC7041095; doi:10.1186/s12879-020-4896-0)
Supplement: Supplementary file 1 — Additional file 1: Table A1. Messages sent to participants in each treatment group. [file 12879_2020_4896_MOESM1_ESM.docx]

**TITLE**: A randomized controlled trial study on the acceptability, feasibility, and preliminary impact of SITA (**S**MS as an **I**ncentive **T**o **A**dhere): A mobile technology-based intervention informed by behavioral economics to improve ART adherence among youth in Uganda

**AUTHORS**: Sarah MacCarthy^[[1]](#footnote-1)^, Zachary Wagner^[[2]](#footnote-2)^, Alexandra Mendoza-Graf^[[3]](#footnote-3)^, Carlos Ignacio Gutierrez3, Clare Samba^[[4]](#footnote-4)^, Josephine Birungi4, Stephen Okoboi^[[5]](#footnote-5)^, Sebastian Linnemayr2

**SUPPLEMENTARY APPENDIX**

Table A1. Messages sent to participants in each treatment group

| Table A1. Messages sent to participants in each treatment group | |
| --- | --- |
| Treatment Group 1 | “Dear XXX, keep up the good work! You made 83%.”  “XXX you have made it to 83%. Great job!”  “Hey XXX you scored 83%. Keep the move on!”  “Good work dear XXX. You did 83%. There is light ahead!” |
| Treatment Group 2 | “Good work dear XXX. 83% compared to many of your SITA buddies at 90%. There is light ahead!”  “Hey XXX you scored 83%. Many guys at SITA scored 90%. Keep the move on!”  “Dear XXX, keep up the good work! You made 83%, others in SITA got 90%.”  “XXX you have made it to 83%. The rest of SITA has a score of 90%. Great job!” |

1. Behavioral and Policy Sciences, RAND Corporation, Santa Monica, CA, USA [↑](#footnote-ref-1)
2. Economics, Sociology, and Statistics, RAND Corporation, Santa Monica, CA, USA [↑](#footnote-ref-2)
3. Pardee RAND Graduate School, Santa Monica, CA, USA [↑](#footnote-ref-3)
4. TASO Uganda, Kampala, Uganda [↑](#footnote-ref-4)
5. Infectious Diseases Institute, Makerere University Kampala, Uganda [↑](#footnote-ref-5)
